# Talking the talk in junior interprofessional education: is healthcare terminology a barrier or facilitator?

**DOI:** 10.1186/s12909-021-02564-4

**Published:** 2021-03-22

**Authors:** Shamara Nadarajah, Arden Azim, Derya Uzelli Yılmaz, Matthew Sibbald

**Affiliations:** 1 Centre for Simulation-Based Learning, Faculty of Health Sciences, McMaster University, 1280 Main St W, Hamilton, ON L8S 4K1 Canada; 2 Department of Medicine, Faculty of Health Sciences, McMaster University, Hamilton, Canada; 3 Izmir Katip Celebi University, Faculty of Health Sciences, Department of Nursing Izmir, Turkey; 4 The McMaster Education Research, Innovation and Theory (MERIT) Program, Faculty of Health Sciences, McMaster University, Hamilton, Canada

**Keywords:** Healthcare terminology, Interprofessional collaboration, Interprofessional education, Jargon, Simulation

## Abstract

**Background:**

Use of healthcare terminology is a potential barrier to interprofessional education (IPE). This study describes how junior learners perceive and classify healthcare terminology in IPE settings.

**Methods:**

We conducted a mixed methods study involving 29 medical, 14 nursing, and 2 physician assistant students who had previously attended or were registered to participate in educational activities at McMaster University’s Centre for Simulation-Based Learning. 23 participants identified “inclusive” or “exclusive” terminology in a series of scenarios used for IPE workshops using an online survey. We collated lists of “inclusive” and “exclusive” terminology from survey responses, and characterized the frequencies of included words. 22 students participated in focus group discussions on attitudes and perceptions around healthcare terminology after attending IPE workshops. We identified themes through an iterative direct content analysis of verbatim transcripts.

**Results:**

Students analyzed 14 cases, identifying on average 21 terms per case as healthcare terminology (28% of overall word count). Of the 290 terms identified, 113 terms were classified as healthcare terminology, 46 as inclusive and 17 as exclusive by > 50% of participants. Analysis of focus group transcripts revealed 4 themes: abbreviations were commonly perceived as complex terminology, lack of familiarity with terminology was often attributed to inexperience, simulation was considered a safe space for learning terminology, and learning terminology was a valued IPE objective.

**Conclusions:**

While students perceive a lot of healthcare terminology in IPE learning materials, categorization of terminology as “inclusive” or “exclusive” is inconsistent. Moreover, healthcare terminology is perceived as a desirable difficulty among junior learners, and should not be avoided in IPE.

**Supplementary Information:**

The online version contains supplementary material available at 10.1186/s12909-021-02564-4.

## Background

Interprofessional education (IPE) involves individuals from multiple health professions learning “about, from, and with each other to enable effective collaboration and improve health outcomes” [1]. Among the many challenges associated with bringing individuals with different educational backgrounds together, use of jargon and complex healthcare terminology has been cited as a potential barrier to IPE [2–4]. In fact, healthcare terminology was identified as one of 10 key challenges of IPE in a recent large systematic review [4]. In response, frameworks for IPE emphasize the need for shared language between professions to optimize communication [5, 6].

Several studies have identified terminology – particularly terminology not easily understood by learners (e.g. acronyms and medication abbreviations) – as a detractor from learning [7, 8] and barrier to effective interprofessional collaboration [9, 10]. Our own research around IPE workshops identified concern that learning materials excluded students with less advanced domain knowledge [11]. Asking for clarification of confusing or unfamiliar terminology can be time consuming [12]. Furthermore, it can impact the way that healthcare professionals understand and relate to one another and work towards a unified goal [3, 13].

Healthcare terminology can be especially challenging for pre-licensure trainees. These learners have little knowledge of healthcare terminology due to their limited workplace exposure. They are often less experienced in the healthcare field and may not have a fully formed professional identity [14]. Language is critical to socialization within any community [15] and an important component of professionalization and role formation [16–18]. This makes it particularly important to consider how healthcare terminology is integrated into interprofessional learning for these learners.

Past studies labelled particular types of healthcare terminology as jargon, a term with a clearly negative connotation, referring to technical sublanguage not well-recognized by out-group members [19–21]. However, an alternate approach involves categorizing healthcare terminology into two distinct subsets: language shared by multiple healthcare professions (“inclusive”), and language unique to specific healthcare professions (“exclusive”). Exclusive language is more often cited as an IPE challenge than inclusive language [2, 3, 7, 8, 12]. For example, during a program evaluation of the Leicester model of IPE, researchers observed that nursing and social work students struggled with “exclusive” terms geared towards medical students such as ‘management plan’ and ‘history taking’ [7]. Another study found that during a series of IPE sessions for nursing and medical students, jargons, such as nursing qualification acronyms, were a source of confusion [2]. A third study aimed at reducing profession-specific terminology among speech-language pathologists identified terms such as ‘articulation’, ‘graphemes’, ‘intelligible’, and ‘orthography’ as exclusive language that hindered collaboration and communication with educators in school communities [12]. In response to this literature, several IPE guides and handbooks warn against the heavy use of discipline-specific language, as it can be a barrier to interprofessional interactions and could impair trainees’ ability to clarify their professional roles effectively [22, 23].

Despite the literature focus on the harms of exclusive language, even inclusive language could be a barrier to learning for pre-licensure trainees in the early stages of training. Students participating in IPE sessions may not all be in the same year of training and do not share the same profession-specific curricula. Profession-specific curricula may introduce different vocabulary at different stages of training, impairing the ability of these junior trainees to find common ground to communicate, collaborate and define their professional roles relative to each other.

This study focuses on understanding how pre-licensure trainees perceive terminology when they encounter it in their early IPE experiences. We conducted a mixed-methods study to explore the following research questions:

1. To what extent is healthcare language reliably identified and classified as “inclusive” or “exclusive” by pre-licensure medical, nursing, and physician assistant students, in IPE learning materials?
2. How is healthcare terminology perceived and valued by pre-licensure healthcare students in an IPE setting?

## Methods

### Participants and recruitment

We recruited 45 pre-licensure students from the undergraduate medical education program (MD students), nursing program (RN students), and physician assistant program (PA students) to participate in either an online survey (part 1) or focus group (part 2).

In part 1, MD, RN, and PA students who had previously participated in educational activities at McMaster University’s Centre for Simulation-Based Learning (CSBL), were recruited via e-mail to participate in a 15–30 min online survey. Students who completed the survey received a $10 gift card.

In part 2, MD, RN, and PA students who were registered to participate in a simulation case-based IPE workshop at the CSBL were asked to participate in a 1-h post-workshop focus group session following the workshop. Participation in the study was optional and had no impact on their experience within the workshop itself.

### Content delivery and data collection

In part 1, participants were e-mailed a survey comprised of 14 written scenarios taken from the CSBL’s existing IPE courseware. The material came from recurring workshops held at the CSBL for the last 3 years, and were created by developers from different backgrounds, including educators, healthcare professionals, and trainees. Minor revisions were made from year to year by educational facilitators, but all revisions pre-existed the study, with no details changed for the purpose of the study. The survey was pilot tested by four CSBL staff members from different professional backgrounds (educators, healthcare workers, and trainee), to confirm the questions were understandable, no training was required for use, and that the task was feasible in a short time frame. The scenarios encompassed a variety of clinical settings and disciplines (Table 1). Participants were provided clear definitions of healthcare terminology and its two subgroups (inclusive and exclusive language), and were prompted to describe their understanding of these terms in their own words. Then, they were asked to (a) highlight all words they considered to be “inclusive language”, and (b) circle all words that they perceived to be “exclusive language”. Participants’ responses were anonymized and compiled.

**Table 1:** Classification of scenarios contained in online survey

Scenario	Discipline
1	Cardiology
2	Cardiology
3	Post-Operative Orthopedics
4	Internal Medicine
5	Pediatrics
6	Obstetrics
7	Geriatrics
8	Geriatrics
9	Internal Medicine
10	Internal Medicine
11	Cardiology
12	Emergency Medicine
13	Obstetrics
14	Endocrinology

In part 2, MD, RN, and PA students attended an optional 2-h case-based IPE workshop, pre-existing this study, anchored around the CIHC framework, related to either transitions to workplace learning or handover of care. The workshops were developed by a team of faculty educators and students from both undergraduate nursing and medicine. The workshops were offered to all RN, MD, and PA students. At the end of each workshop, students were recruited to a 1-h focus group in order to better understand perceptions and attitudes surrounding healthcare terminology and jargon. In total, two focus group discussions were conducted. The focus groups were facilitated by three members of the research team (A.A. posed questions, while S.N. and D.U. took notes), and were semi-structured, guided by open-ended questions. Focus group discussions were recorded and transcribed verbatim, and participant’s names were replaced with non-identifying codes.

### Data analysis

We used the survey responses to collate all healthcare terms identified, as well as the frequency with which they were identified by participants. Frequencies of coded words were tabulated across professional subgroups, combining MD and PA students as they share a common pre-clinical curriculum. Finally, we calculated the percentage of words coded as inclusive and exclusive. Focus group transcripts were qualitatively analyzed using content analysis, informed by the Colaizzi method [24, 25]. Three investigators from different professional backgrounds independently read and reread all transcripts identifying key themes using a constant comparative approach. We finalized themes through two consensus discussion sessions where we also identified key quotes. After consolidation of the themes, we performed member checking with a group of 9 students with representation from each subgroup (MD, RN, and PA students) as described previously [26].

### Ethics

The study was approved by the Hamilton Integrated Research Ethics Board on October 15, 2019 (project #7821). Informed written consent was obtained from all participants.

## Results

### Part 1: online survey

**Fig. 1:**
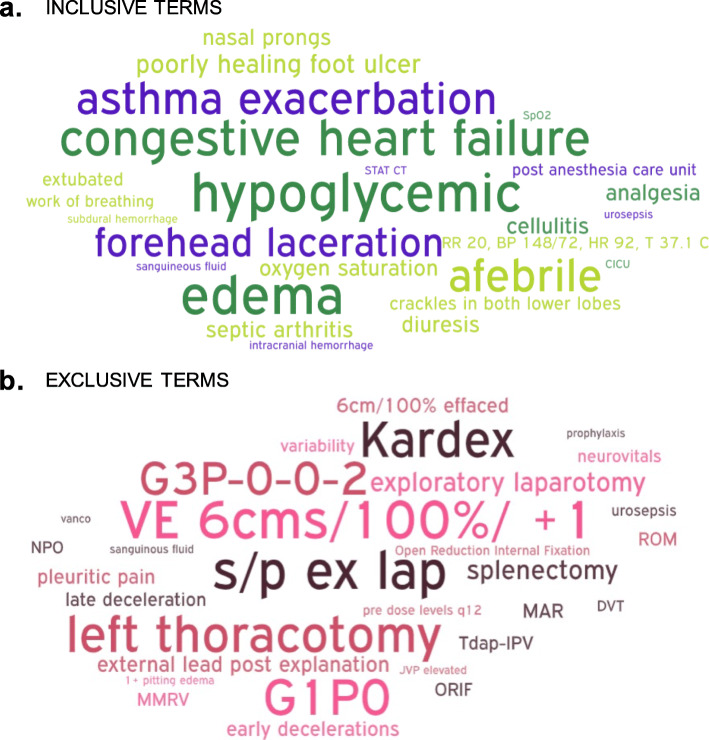
Word cloud of top inclusive (**a**) and exclusive (**b**) terms. Larger font size represents a greater frequency of categorization of a given word as exclusive or inclusive by the study participants

**Fig. 2:**
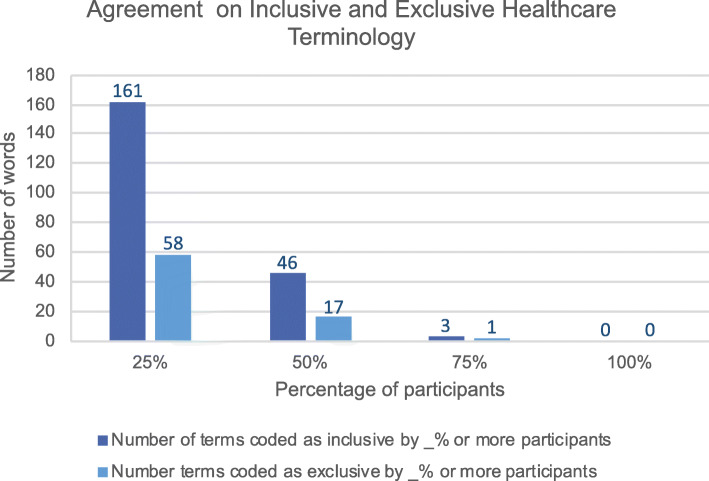
Agreement between participants on inclusivity or exclusivity of terms. Shown are the number of terms classified as inclusive or exclusive by 25% or more, 50% or more, and 75% or more participants. No term was classified by all participants as either inclusive or exclusive

23 students (15 MD, 7 RN, 1 PA) analyzed 14 IPE workshop cases covering a range of healthcare settings and disciplines (Table 2). Terms coded as inclusive and exclusive healthcare terminology were graphically represented in word clouds (Fig. 1). Students identified 290 healthcare terms (28% of total word count), with 285 coded as inclusive, and 196 coded as exclusive by at least one participant (Table 3). Only 4 terms were classified as healthcare terminology by all participants, and no terms were classified unanimously as inclusive or exclusive (Tables 4–6 in Appendix). More than 50% of participants classified 113 words as healthcare terminology, 46 as inclusive, and 17 as exclusive (Fig. 2). When RN students were compared with PA and MD students, no differences were noted in the identification or classification of terminology (Tables 4–6 in Appendix).

**Table 2:** Demographic information of online survey and focus group participants

**Category**	**No. of participants (online survey)**	No. of participants (focus group)
**Program**		
MD	15	14
RN	7	7
PA	1	1
**Level/Year of Training**^**a**^		
1	8	14
2	8	1
3	5	6
4	2	1
**Age**		
≤ 21	7	6
22–24	14	13
≥ 25	2	3
**Gender**		
Male	5	4
Female	18	18
**Weeks of Clinical Exposure**		
< 1 week	4	2
1 week – 1 month	3	10
> 1 month	16	10
**Total**	**23**	**22**

^a^Program length varies by program. At McMaster University, the MD program is 3 years, the RN program is 4 years, and PA program is 2 years

**Table 3:** Breakdown of healthcare language identified by at least one participant by case

Case	Total word count	# Healthcare terms^**a**^ identified (% word count)	# Inclusive terms^**a**^ identified (% word count)	# Exclusive terms^**a**^ identified (% word count)
1	212	30 (32)	30 (32)	24 (25)
2	121	22 (45)	22 (45)	18 (40)
3	198	34 (29)	34 (29)	21 (16)
4	133	13 (17)	13 (17)	10 (12)
5	181	14 (17)	13 (14)	8 (9)
6	124	29 (42)	29 (42)	24 (36)
7	227	29 (30)	28 (29)	17 (20)
8	142	32 (38)	30 (37)	27 (32)
9	105	10 (16)	10 (16)	7 (11)
10	126	10 (17)	10 (17)	2 (4)
11	128	15 (18)	14 (18)	7 (10)
12	125	22 (43)	22 (43)	13 (18)
13	108	18 (40)	18 (40)	13 (27)
14	143	12 (15)	12 (15)	5 (7)
**Total**	**2073**	**290**	**285**	**196**
**Average**	**148**	**21 (28)**	**20 (28)**	**14 (19)**

^a^terms may be comprised of more than one word (e.g. oxygen saturation, two-person assist)

### Part 2: focus group

22 students (14 MD, 7 RN, 1 PA) participated in two focus groups (Table 2), sharing both the challenges and benefits of introducing terminology in early IPE experiences. They described their early clinical experiences and made comparisons between these experiences and the current workshop. Four themes emerged.

#### Theme #1 – abbreviations and acronyms as a form of complex healthcare language

When prompted to reflect on their experiences with healthcare language, many participants pointed to the heavy use of abbreviations in the clinical setting. Abbreviations were largely regarded as a barrier to interprofessional and interdisciplinary communication, particularly when the same abbreviation had different meanings in different settings. Rather than improving the efficiency of communication, students felt that abbreviations increased the amount of time spent deciphering and interpreting the term correctly.

*One of the (cases) said ROM … I was like, "Oh, that's probably ruptured membranes," but, is it? And having to take the time to search the rest of the chart data, to see if that's what I'm thinking it is, probably takes longer than someone just actually writing out ruptured membranes.*

– RN student

*I don’t remember what it was, but it was a three letter kind of thing. And to the nursing students it meant one thing and to the medical students it meant a completely different thing, but it was the exact same three letters. It’s really interesting that we have things like that, and for people that work so close together, that we have such confusing terminology … it obviously can lead to a lot bigger issues.*

– RN student

#### Theme #2 – unfamiliar terminology: product of being an early learner or exclusionary?

Participants found it challenging to label terms exclusive as they were unsure whether their unfamiliarity was due to a lack of clinical experience or lack of use of the terms by their profession.

*One specific example I could think of was about the MAR... I'm only three months into my program, so I have no idea what it was, but it was interesting hearing how much each type of professional used it, and under what context they did.*

– MD student

Two individuals commented on the value of encountering exclusive terminology in early IPE experiences, explaining that if terms – even exclusive terms – are used in the workplace, then knowing them was helpful.

*I think as long as it's continued in hospital … until it's fully changed I think it is beneficial to have here because realistically, as it will continue to be used in hospital, it does help to put this into clinical setting now, whether we fully believe if it should be used or not long term.*

– Unknown profession

#### Theme #3 – simulation as a safe space

Participants noted that simulation was a safe setting to learn terminology. It was easier to ask for help from facilitators and peers compared to the clinical setting. They acknowledged that the sessions were focused on their learning, without the pressure of patient care responsibilities.

*This is a learning space. I think one of the facilitators actually mentioned that explicitly. She was like "This is a safe space. You're not expected to know everything and that's okay, just ask."*

– RN student

Participants drew comparisons to clinical settings where they commonly encountered unfamiliar terminology but found it difficult to ask for help, due to the fast-pace and perception that questions might adversely impact their assessment. As a result, students were more likely to conduct an internet search to learn terminology in the workplace, rather than asking for help.

*A lot of times during horizontals, you don't get the chance to ask questions, because everyone's really busy with their own thing. So I think it's really helpful to learn about it in more of an academic setting as opposed to a clinical setting.*

– MD student

#### Theme #4 – value of complex terminology as a desirable difficulty in early IPE

Participants pointed out the additional cognitive effort required in integrating healthcare terminology, and the ways in which it could challenge learning.

*I just felt like it took me a lot of effort to read everything, internalize it, and then regurgitate it. And then to have to decipher it on top of all that.*

– PA student

However, participants pointed out specific benefits of utilizing healthcare terminology in early IPE experiences. They appreciated that cases reflected authentic clinical experiences.

*I definitely don't think that the cases should be dumbed down, even though I'm in first year and I don't know a lot, but it's just because, they weren't so complicated that we couldn't get the big picture, or the main points at all. Even though there are some words that we didn't know, the big picture is very clear in each case, and because we read these cases, thought about it, and thought about how to do the hand-over in this slightly stressful situation, I don't think I'm gonna forget any of the new terms I learned here. So I think it's actually beneficial to have it a little bit more advanced than what we're learning in school.*

– MD student

Participants believed that healthcare terminology should be introduced as early as possible, even prior to clinical exposure.

*I actually think that people should do it as many times throughout their schooling as possible, because what you get in first year is gonna be very different than what you get in second year … it's gonna be very, very different, what you're able to take away and maybe contribute to other members in your group of varying learning levels as well.*

– PA student

## Discussion

Through our study, we explored two types of healthcare language: language inclusive of different professions, and language exclusive to one profession. Our mixed method approach allowed us to explore these constructs in a robust manner. We found that healthcare terminology in IPE educational materials was easily identified by pre-licensure trainees, although they frequently disagreed about whether it was inclusive or exclusive. Focus group discussions revealed that, although challenging, students valued encountering healthcare terminology early in their training, particularly in the interprofessional setting. They emphasized the importance of a safe and comfortable environment to facilitate learning. Our study has broad implications for both researchers and educators in the field of pre-licensure trainee IPE.

The inconsistencies noted in categorizing healthcare language among undergraduate healthcare students may relate to individual differences in depth and length of prior clinical exposure, or systemic differences in curriculum across training programs. It is possible that jargon is part of the hidden curriculum [27], becoming more recognizable over time as individuals socialize into their respective professions, and identify more easily their in-group versus out-group language. Nevertheless, focus group participants demonstrated a strong grasp of the constructs of inclusive versus exclusive language, and of the potential detrimental impact of exclusive terminology on workplace collaboration. Recognition of terminology was often perceived as a ‘desirable difficulty’ of interprofessional learning. While the words themselves are identified as inclusive or exclusive during learning, they blend into regular lexicon as students spend more time learning in an interprofessional environment.

A key theme that emerged was the value of learning terminology in a “safe space”. Elective simulation opportunities were recognized as unique spaces where students can learn terminology with more opportunity than workplace settings to clarify ambiguous or novel terms, and less perceived identity and social risk. Other studies have highlighted the benefits of using simulation to optimize learning and described conditions that maximize learner safety, such as approachable and accessible facilitators, normalizing mistakes, pre-briefing, and mandating that learners respect each other [28–31]. Many (if not all) of these conditions were present in the workshops we studied. Participants commented that facilitators were approachable and helped learners decipher challenging language. The workshops were entirely voluntary and separate from each program’s curriculum and evaluation, allowing students to feel comfortable making errors. Pre-workshop briefings gave students the necessary context to participate in the workshop, without the need to rely on prior knowledge. Finally, students agreed to a learning contract prior to participation in the workshop, which acted to preserve the psychological wellbeing of learners when asking questions.

Use of exclusive language is a challenge not only in IPE settings, but also in the workplace [32, 33]. Systematic reinforcement of exclusive language occurs with junior trainees feeling the need to know and understand exclusionary jargon, despite the implications of its usage for effective interprofessional collaboration. A study that surveyed dentistry students and faculty members found that 71% of participants learned jargon from their colleagues, and 38% learned jargon from their teachers, much of which involved non-standard abbreviations and terminology [34]. This suggests that the use of exclusive language is commonplace in the clinical setting, and can be passed colleague to colleague. In this study and others [35, 36], students commented that exclusive terminology was worth learning through IPE workshops, because whether or not the terminology is appropriate, they are required to understand it in the workplace as it is used by others. They appreciated the opportunity to have discussions surrounding the usage and underlying meaning of exclusive terms.

Given this data, how should the educator approach healthcare terminology in IPE? One way is to treat healthcare terminology as an unwanted barrier or challenge that should be reduced. In the past educators have rewritten material that they felt contained confusing or exclusive terminologies [7]. However, our work lays foundation for another approach, which is to treat terminology as a learning objective in and of itself, and to allow time and facilitator guidance for students to decipher and learn the terminology themselves. In our study, students valued the opportunity to learn new vocabulary in a safe environment and did not want the scenarios to be simplified. They felt that the additional cognitive workload associated with learning terminology was manageable, and they were able to absorb terminology while simultaneously focusing on the workshop’s other learning objectives. They relied on one another and facilitators to learn this terminology collaboratively – ironically, this became a catalyst for true interprofessional learning in our workshops. Engineering this catalytic effect requires workshop participants to have varied types of workplace exposures, allowing reliance on each other’s unique experiences to decipher many unfamiliar terms.

In determining a framework to mitigate the challenges of terminology in IPE, we propose a spectrum of corrective action, where the intervention varies depending on contextual factors. On one end of the spectrum would be to remove all healthcare terminology from IPE learning materials, such as changing ‘hypoglycemic’ (see Table 6 in Appendix) to ‘low blood sugar’ and ‘cellulitis’ (see Table 6 in Appendix) to ‘skin infection’. Another method would be to provide the definitions of terminologies in advance [4]. On the other end of the spectrum, healthcare terminology would be treated as its own learning objective in addition to the session’s existing goals, within a safe environment for students to learn terminology from interprofessional peers. The approach taken should be dependent on the participants, facilitators, and nature of the session’s objectives. Our study provides an example of how the latter approach can be well-received and effective in promoting interprofessional learning.

### Limitations

Through this study, we were able to gather data from trainees of three different healthcare programs. However, participation was voluntary with disproportionate female and MD students enrolling. As a result, we acknowledge the results may be skewed by this sample, particularly as participants may have more favorable impressions of interprofessional education than their peers. Furthermore, we cannot comment on perceptions of other allied health professionals such as occupational therapists, physiotherapists, pharmacists, and social workers, nor can we comment on changes in perception at different stages of training. Finally, the study was conducted in a single tertiary care institution, and cannot be extrapolated to different healthcare contexts, particularly where multiple different languages are used.

## Conclusion

In this study, undergraduate trainees easily engaged in a conversation around healthcare terminology with facile understanding of the constructs of inclusive and exclusive language. These discussions were well-received even at an early stage of training. Elective simulation opportunities were perceived as unique spaces where students can learn the terminology with more opportunity to clarify ambiguous or novel terms than workplace settings.

Existing literature that cautions against the use of healthcare terminology in IPE may need nuancing. Many students perceived healthcare terminology as a valued learning objective and facilitator of IPE. For educators we recommend: (1) making terminology a learning objective—this was well-received by students who recognize this as a genuine workplace challenge and not an artefact of educational materials; and (2) worrying less about whether terminology is perceived as inclusive or exclusive and focusing instead on creating a safe and inclusive environment conducive to learning the healthcare terminology. For researchers, we recommend: (1) studying how healthcare terminology perceptions change through the trajectory of training and (2) identifying the impact of terminology in sensitive healthcare processes such as handovers and transitions.

In summary, including healthcare terminology in IPE can prompt students to learn with, from, and about one another, achieving the ultimate goal of IPE.

### Supplementary Information

**Additional file 1.**

## Data Availability

The datasets during and/or analysed during the current study available from the corresponding author on reasonable request.

## Appendix

**Table 4:** List of Top 20 Healthcare Terms (and Ties)

Term	Frequency Coded as Healthcare Terminology		
	Overall (*n* = 23)	RN (*n* = 7)	MD/PA (*n* = 16)
left thoracotomy	1.00	1.00	1.00
VE 6cms/100%/ + 1	1.00	1.00	1.00
urosepsis	1.00	1.00	1.00
pleuritic pain	1.00	1.00	1.00
DVT	0.96	1.00	0.94
Tdap-IPV and MMRV	0.96	0.86	1.00
Kardex	0.96	0.86	1.00
s/p ex lap	0.96	1.00	0.94
exploratory laparotomy	0.96	0.86	1.00
splenectomy	0.96	1.00	0.94
sanguineous fluid	0.96	1.00	0.94
diuresis	0.96	1.00	0.94
G1P0	0.91	0.86	0.94
NPO	0.91	0.86	0.94
prophylaxis	0.91	0.86	0.94
STAT CT	0.91	0.71	1.00
cellulitis	0.91	1.00	0.88
hypoglycemic	0.91	1.00	0.88
edema	0.91	1.00	0.88
G3P-0-0-2	0.91	0.71	1.00
late deceleration	0.91	0.86	0.93

**Table 5:** List of Top 20 Exclusive Terms

Term	Frequency Coded as Exclusive		
	Overall (*n =* 23)	RN (*n =* 7)	MD/PA (*n =* 16)
s/p ex lap	0.78	1.00	0.69
VE 6cms/100%/ + 1	0.77	1.00	0.67
left thoracotomy	0.70	0.86	0.63
Kardex	0.70	0.43	0.81
G1P0	0.70	0.86	0.63
G3P-0-0-2	0.64	0.71	0.60
exploratory laparotomy	0.61	0.57	0.63
splenectomy	0.61	0.86	0.50
external lead post explanation	0.59	0.71	0.53
pleuritic pain	0.57	0.57	0.56
MAR	0.57	0.71	0.50
6 cm/100% effaced	0.57	0.71	0.50
early decelerations	0.57	0.71	0.50
ORIF	0.52	0.29	0.63
Tdap-IPV and MMRV	0.52	0.57	0.50
ROM	0.52	0.71	0.44
late deceleration	0.52	0.71	0.44
variability	0.50	0.71	0.40
NPO	0.48	0.86	0.31
neurovitals	0.48	0.57	0.44

**Table 6:** List of Top 20 Inclusive Terms

Term	Frequency Coded as Inclusive		
	Overall (*n =* 23)	RN (*n =* 7)	MD/PA (*n =* 16)
hypoglycemic	0.78	0.71	0.81
edema	0.78	0.86	0.75
congestive heart failure	0.78	0.86	0.75
afebrile	0.74	0.57	0.81
asthma exacerbation	0.74	0.71	0.75
forehead laceration	0.70	0.71	0.69
poorly healing foot ulcer	0.68	0.86	0.60
diuresis	0.65	0.57	0.69
cellulitis	0.65	0.57	0.69
septic arthritis	0.65	0.57	0.69
nasal prongs	0.65	0.43	0.75
analgesia	0.65	0.43	0.75
oxygen saturation	0.65	0.57	0.69
extubated	0.61	0.43	0.69
crackles in lower lobes	0.61	0.57	0.63
post anesthesia care unit	0.61	0.43	0.69
work of breathing	0.61	0.43	0.69
RR 20 BP 148/78, HR 92, T 37.1 C	0.61	0.57	0.63
SpO2	0.61	0.43	0.69
urosepsis	0.61	0.57	0.63

## Abbreviations

IPE: Interprofessional education
CIHC: Canadian Interprofessional Health Collaborative
MD: Medical doctor
RN: Registered nurse
PA: Physician assistant
CSBL: Centre for Simulation-Based Learning
