# Supplementary material for: Talking the talk in junior interprofessional education: is healthcare terminology a barrier or facilitator?
Source: BMC Med Educ. 2021 Mar 22;21:177. doi: 10.1186/s12909-021-02564-4 (PMC7986253; doi:10.1186/s12909-021-02564-4)
Supplement: Supplementary file 1 — Additional file 1. [file 12909_2021_2564_MOESM1_ESM.docx]

**SUPPLEMENTARY MATERIAL**

**S1. Sample mock-up of survey case by study participant.** Survey participants were emailed a form containing 14 cases, an example of which is provided here. Participants were asked to highlight words they considered “inclusive” language and circle words they considered “exclusive” language.

Mary Smith, DOB 3-7-82, a healthy female G1P0 patient has been admitted to the delivery room in active labor, initially with contractions q5min. She denies ROM or vaginal bleeding. She is at 39-weeks gestation. She is placed on a monitor, with her call light within reach. She has had excellent prenatal care and no complications. She is currently dilated to 6 cm/100% effaced, but her contractions have lessened in intensity and frequency. Her fetal heart rate rhythm strip shows baseline 140 BPM, some early decelerations, and one late deceleration with good variability that returns to baseline. She is afebrile, with normal vitals. Her membranes ruptured 12 hours ago, so there is some urgency to keep her labor progressing in order to avoid a C-Section.
